# New CRISPR Mutagenesis Strategies Reveal Variation in Repair Mechanisms among Fungi

**DOI:** 10.1128/mSphere.00154-18

**Published:** 2018-04-25

**Authors:** Valmik K. Vyas, G. Guy Bushkin, Douglas A. Bernstein, Matthew A. Getz, Magdalena Sewastianik, M. Inmaculada Barrasa, David P. Bartel, Gerald R. Fink

**Affiliations:** a Whitehead Institute for Biomedical Research, Cambridge, Massachusetts, USA; b Department of Biology, Massachusetts Institute of Technology, Cambridge, Massachusetts, USA; c Department of Biology, Ball State University, Muncie, Indiana, USA; d Howard Hughes Medical Institute, Cambridge, Massachusetts, USA; Carnegie Mellon University

**Keywords:** CRISPR, *Candida*, *Naumovozyma*, *Saccharomyces*, *albicans*, *castellii*, *cerevisiae*, *glabrata*

## Abstract

We have created new vectors for clustered regularly interspaced short palindromic repeat (CRISPR) mutagenesis in Candida albicans, Saccharomyces cerevisiae, *Candida glabrata*, and Naumovozyma castellii. These new vectors permit a comparison of the requirements for CRISPR mutagenesis in each of these species and reveal different dependencies for repair of the Cas9 double-stranded break. Both C. albicans and S. cerevisiae rely heavily on homology-directed repair, whereas C. glabrata and N. castellii use both homology-directed and nonhomologous end-joining pathways. The high efficiency of these vectors permits the creation of unmarked deletions in each of these species and the recycling of the dominant selection marker for serial mutagenesis in prototrophs. A further refinement, represented by the "Unified" Solo vectors, incorporates Cas9, guide RNA, and repair template into a single vector, thus enabling the creation of vector libraries for pooled screens. To facilitate the design of such libraries, we have identified guide sequences for each of these species with updated guide selection algorithms.

**IMPORTANCE** CRISPR-mediated genome engineering technologies have revolutionized genetic studies in a wide range of organisms. Here we describe new vectors and guide sequences for CRISPR mutagenesis in the important human fungal pathogens C. albicans and C. glabrata, as well as in the related yeasts S. cerevisiae and N. castellii. The design of these vectors enables efficient serial mutagenesis in each of these species by leaving few, if any, exogenous sequences in the genome. In addition, we describe strategies for the creation of unmarked deletions in each of these species and vector designs that permit the creation of vector libraries for pooled screens. These tools and strategies promise to advance genetic engineering of these medically and industrially important species.

## Abstract (precis)

CRISPR-mediated genome engineering technologies have revolutionized genetic studies in a wide range of organisms. Here we describe new vectors and guide sequences for CRISPR mutagenesis in the important human fungal pathogens C. albicans and C. glabrata, as well as in the related yeasts S. cerevisiae and N. castellii. The design of these vectors enables efficient serial mutagenesis in each of these species by leaving few, if any, exogenous sequences in the genome. In addition, we describe strategies for the creation of unmarked deletions in each of these species and vector designs that permit the creation of vector libraries for pooled screens. These tools and strategies promise to advance genetic engineering of these medically and industrially important species.

## INTRODUCTION

Advances in clustered regularly interspaced short palindromic repeat (CRISPR) technology have greatly increased the ability to manipulate the genomes and gene expression of a wide variety of organisms (1–3). CRISPR-based mutagenesis requires a nuclease (usually Cas9) and an appropriate RNA guide targeted to the gene of interest; however, the outcome of CRISPR mutagenesis may depend on the idiosyncratic biology of the organism, especially with respect to its specific DNA repair system. The majority of systems utilize plasmid or viral expression vectors to introduce *CAS9* and RNA guides into the organism, although some researchers have successfully used purified RNA/Cas9 protein complexes (4–8). The guide RNA directs the Cas9 nuclease to the complementary genomic sequence, where it makes a double-stranded break (DSB). Either the nonhomologous end-joining (NHEJ) pathway or the homology-directed repair (HDR) pathway repairs these cuts. NHEJ joins nonhomologous ends of DNA and represents the most common method of repair of CRISPR DSBs in mammals (5, 6). During NHEJ, bases are often added or deleted from the ends of DSBs, resulting in insertions or deletions (indels) in the targeted locus. When the HDR pathway is used, repairs using templates from the homologous chromosome restore the wild-type (WT) sequence. For CRISPR-mediated HDR, exogenous repair templates with homology flanking the DSBs can be used to introduce precise mutations into the genome.

Recently, we developed a CRISPR system that efficiently edits the human fungal pathogen Candida albicans (9). This system has subsequently undergone considerable optimization, and newer guide expression methods have improved the mutagenesis efficiency (10). Transient expression approaches have also permitted efficient deletion using drug resistance markers (11–13). In addition to the targeting sequence and nuclease, CRISPR mutagenesis in C. albicans and its distant relative Saccharomyces cerevisiae requires the inclusion of a repair template, as DSBs are primarily repaired by HDR (7, 9). Conservation of this requirement for a repair template among other fungi is unknown.

To explore the requirement for a repair template, we designed a number of improved vectors for CRISPR-mediated genome editing in S. cerevisiae and C. albicans as well as in two other fungi, C. glabrata and Naumovozyma castellii. Our recyclable constructs use the dominant nourseothricin resistance (Nat^r^) drug resistance marker and are able to generate unmarked kilobase-scale deletions with a single guide sequence when coupled with a repair template. Although efficient mutagenesis in C. albicans and S. cerevisiae requires the addition of a repair template, C. glabrata and N. castellii require only *CAS9* and a guide RNA. These results suggest that HDR is the predominant repair pathway in S. cerevisiae and C. albicans, in contrast to two close relatives of S. cerevisiae, N. castellii and C. glabrata, in which the NHEJ pathway dominates. Nevertheless, mutations of *RAD* genes that disable the HDR system bypass repair template requirements in S. cerevisiae, leading to repair via NHEJ. However, mutations in the homologous *RAD* genes do not bypass the repair template requirements in C. albicans, which suggests that S. cerevisiae and C. albicans have distinct repair preferences. In addition, we demonstrate that CRISPR can be used to generate loss-of-heterozygosity (LOH) events in C. albicans in the absence of exogenous repair templates.

To circumvent the requirement for cotransformation of both a Cas9/single guide RNA (sgRNA) plasmid and a repair template, we developed Unified Solo vectors, which consolidate repair templates onto the Cas9/sgRNA delivery plasmid. This consolidation enables pooled mutagenesis screening in any given background, including in diverse clinical isolates. In addition, using updated guide selection algorithms, we have designed guides for each of the aforementioned species and found that most genes (>90%) can be specifically modified by CRISPR. The combination of these improved vectors with updated guide selection algorithms sets the stage for the development of inclusive genome-wide mutant libraries in each of these species.

## RESULTS

### Improved vectors for mutagenesis in C. albicans*.*

**FIG 1:**
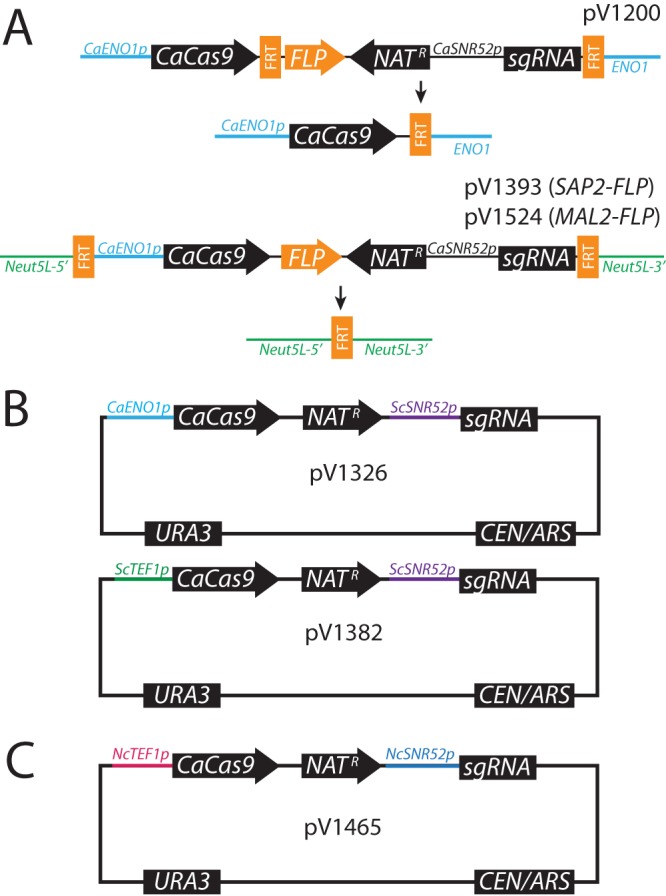
Vectors for CRISPR mutagenesis in Candida albicans, *Candida glabrata*, Naumovozyma castellii, and Saccharomyces cerevisiae. (A) Recyclable C. albicans CRISPR vector pV1200 (previous generation) replaces one copy of *ENO1*, and flipout removes *NAT^r^* and *sgRNA* gene sequences. Current vectors pV1393 and pV1524 insert into the *Neut5L* locus, and flipout leaves only an FRT insertion at *Neut5L*. Vector pV1393 uses *SAP2p* to drive *FLP*, while pV1524 uses *MAL2p*. (B) Vectors pV1326 and pV1382 for CRISPR mutagenesis in C. glabrata and S. cerevisiae. (C) Vector pV1465 for CRISPR mutagenesis in N. castellii.

To enable more-flexible CRISPR-based mutagenesis in C. albicans, first, we designed two improved Solo vectors for CRISPR mutagenesis (Fig. 1A, bottom). Previously, Cas9 was integrated into the *ENO1* locus, resulting in a single functional copy of *ENO1* (Fig. 1A, top) (9). As heterozygosity for *ENO1* might affect growth under some conditions (14), our new vectors insert the CRISPR cassette into the *Neut5L* site, a locus whose disruption has been previously reported not to impact growth (15). Second, the previous approach removed only the guide expression module while retaining Cas9. To avoid possible complications of continued Cas9 expression, the FLP recombination target (FRT) sites were relocated to a position flanking the entire cassette such that flippase expression results in complete removal of the cassette, except for a small FRT insertion. Third, we created a variant with flippase expression under control of the inducible *MAL2* gene instead of the *SAP2-FLP* genes because some mutant strains of C. albicans do not robustly induce *SAP2-FLP* expression.

### Markerless deletion mutants.

**FIG 2:**
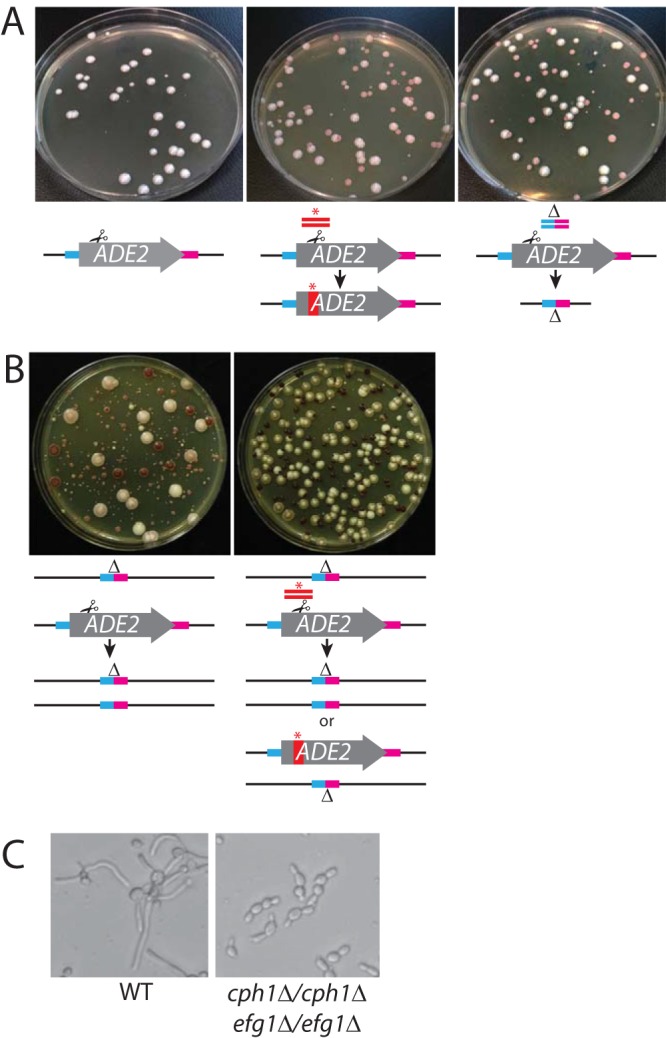
(A) CRISPR mutagenesis in C. albicans to create unmarked deletions and LOH. Strain SC5314 was transformed with *ADE2*-targeting plasmid pV1081 without a repair template (left), with a stop codon repair template (middle), or with a deletion repair template (right). (B) VY841 (*ade2*/*ADE2*) transformed with pV1081 without a repair template (left) or with a stop-codon repair template (right). (C) Serial mutagenesis of SC5314 with pV1393-based vectors used to create *cph1*/*cph1 efg1*/*efg1* mutants. Strains of the indicated genotype were grown overnight in YPD medium and were transferred to RPMI medium–10% serum for 4 h.

The limited number of dominant markers in C. albicans makes the task of creating strains with multiple mutations onerous. To determine whether one could construct unmarked deletions using a single guide sequence, we targeted the *ADE2* locus using a guide that cuts within the open reading frame as previously reported (9) but utilized a repair template that juxtaposed 50 bp upstream of the open reading frame to 50 bp downstream of the *ADE2* open reading frame. This repair template generated *ade2*/*ade2* deletions of 1,652 bp at a high rate comparable to those seen with other *ADE2* repair templates that created only base pair substitutions (61% for deletion versus 100% for stop codon; Fig. 2A). This result means that one can construct multiple unmarked deletion mutations in a strain without complex recycling of the limited dominant markers and without leaving FRT insertion sites at deleted loci.

Using the improved vectors (derived from pV1393 and pV1524), we demonstrated that double mutant strains of C. albicans could be produced with facility. To create a double *cph1Δ*/*cph1Δ;efg1Δ*/*efg1Δ* strain by sequential transformation, we first targeted the *EFG1* gene with a deletion template. Transformants that were *efg1Δ*/*efg1Δ* strains were grown in medium to induce expression of flippase to remove the Cas9 machinery (see Materials and Methods), and flipout clones were then transformed with a vector containing guides and repair templates for deletion of *CPH1*, leading to the isolation of correct double mutant clones (*cph1Δ*/*cph1Δ efg1Δ*/*efg1Δ*; Fig. 2C). The frequency of deletion formation—markerless at both loci—enabled easy construction not only of morphological double mutants but also of auxotrophic double mutants (e.g., *leu2Δ*/*leu2Δ;met15Δ*/*met15Δ*; see Table S2 in the supplemental material) where both mutant sites were free of the marker used to select transformants.

### Unified Solo vectors consolidate Cas9, guide RNA, and repair templates in a single plasmid.

A major limitation to CRISPR mutagenesis in C. albicans is the requirement to provide a separate repair template for efficient mutagenesis. This requirement makes pooled screens with a library of guides virtually impossible, as the likelihood that any given cell will get a matched guide and repair template during transformation diminishes exponentially with increasing library complexity. To circumvent this problem, we created a “Unified” Solo vector that links both guide and repair template on a single molecule.

**FIG 3:**
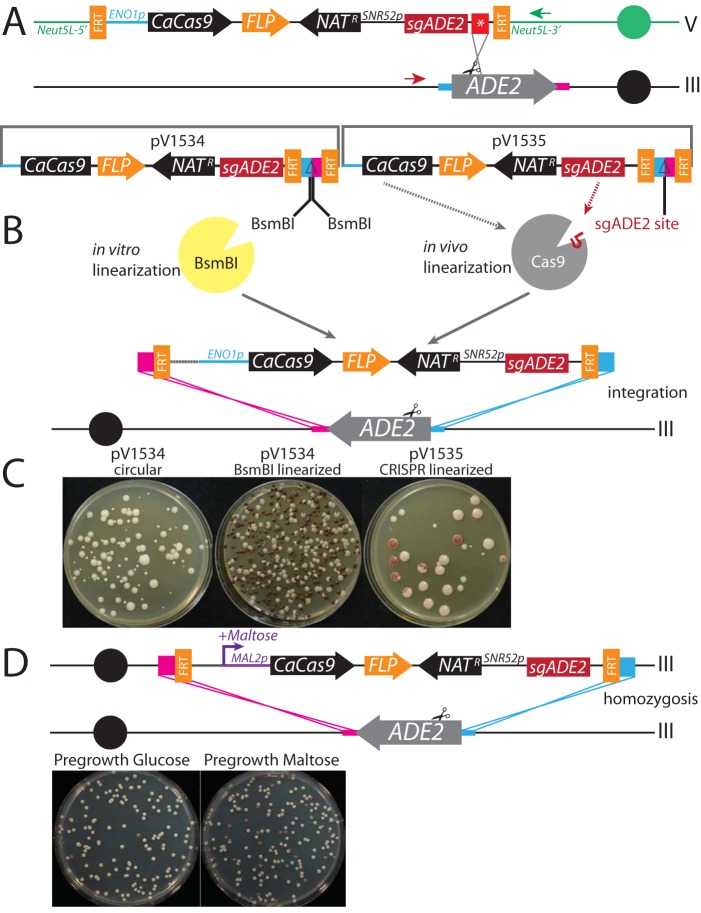
Unified Solo vectors for mutagenesis in C. albicans. (A) Unified Solo vector integrated at *Neut5L* directed against *ADE2* results in translocations detectable by PCR with the indicated primers (red and green arrows). (B) Gene-drive-based Unified Solo vector to target CRISPR to delete *ADE2*. Vector pV1534 is linearized *in vitro* with BsmBI, while vector pV1535 is linearized *in vivo* by CRISPR. (C) Transformation of SC5314 with circular pV1534 (left), BsmBI-linearized pV1534 (middle), or circular pV1535 (right). (D) Maltose-induced gene drive targeting *ADE2*. Heterozygous mutants can be induced to create homozygous mutants by growth in maltose medium.

Our first attempt utilized a stop-codon repair template for *ADE2* (as in Fig. 2A) inserted immediately downstream of the sgRNA terminator (Fig. 3A). This Unified Solo vector was able to mutagenize *ADE2* at a rate comparable to that achieved in our previous Solo system with a separate repair template. However, careful genotyping revealed that all of the *ade2* mutants that we had recovered were the result of a translocation between the *Neut5L* locus (where the CRISPR system that includes the homologous repair template resides) and the *ADE2* locus (Fig. 3A). We observed similar translocations in S. cerevisiae when we provided a repair template that spanned two chromosomal loci (see Text S1 and Fig. S1 in the supplemental material).

10.1128/mSphere.00154-18.2 FIG S1  CRISPR mutagenesis in S. cerevisiae with multiple guides and repair templates. (A) S. cerevisiae strain L6437 was cotransformed with plasmids pV1386 and pGB9 alongside appropriate stop repair templates, as indicated. Transformants displayed either the *ade2* genotype or *leu2* genotype but not the *ade2 leu2* genotype. (B) Strain L6437 was cotransformed with plasmids as described above except that a consolidated repair template was used that linked the stop repair templates described in the panel A legend into a single molecule. While most transformants were either *ade2* or *leu2*, *ade2 leu2* double mutants were identified. (C) PCR genotyping identified sequences consistent with translocation between the *ADE2* and *LEU2* chromosomes. Download FIG S1, PDF file, 0.1 MB. Copyright © 2018 Vyas et al. 2018 Vyas et al. This content is distributed under the terms of the Creative Commons Attribution 4.0 International license.

10.1128/mSphere.00154-18.1 TEXT S1  Targeting multiple genes in one transformation using CRISPR. Download TEXT S1, PDF file, 0.1 MB. Copyright © 2018 Vyas et al. 2018 Vyas et al. This content is distributed under the terms of the Creative Commons Attribution 4.0 International license.

To avoid such chromosome aberrations, which are likely to occur when homologous DNA sequences exist at two or more distinct locations in the genome, we designed a gene-drive-based Unified vector (Fig. 3B), based on a concept presented by DiCarlo et al. (16). Gene drive constructs insert the entire CRISPR system at the locus of the gene to be mutated, rendering homologous regions aligned such that all recombination events are restricted to the locus of interest. To ensure that our gene-drive construct could be inactivated after mutagenesis (for marker recycling and to safeguard against further Cas9 activity), we repositioned the FRT sequences as depicted in Fig. 3B. The repair template for this strategy differs from the previous repair templates in that its homology arms direct insertion of the entire plasmid into the target mutagenesis site, resulting in replacement of the *ADE2* coding region with the Unified vector (Fig. 3B).

As episomal plasmids are not stable in C. albicans, the construct must be linearized to enable integration. We designed two strategies for vector linearization. The first strategy used the BsmBI restriction endonuclease *in vitro*; linearized vector generated *ade2*/*ade2* mutants at a rate of close to 100%, whereas uncut vector failed to produce any mutants (Fig. 3C). The second strategy substituted the BsmBI sites with a target site for the sgADE2 guide and relied on Cas9/sgADE2-mediated cleavage *in vivo* of the circular plasmid at that target site. That strategy also produced *ade2*/*ade2* mutants at a frequency of close to 100%, while eliminating the need to linearize the plasmid before transformation (Fig. 3B).

We reasoned that if gene-drive-mediated homozygosis requires Cas9 expression, it could be modified to create conditional knockouts. To test this, the constitutive *ENO1* promoter in the Unified vector was replaced with the maltose-inducible *MAL2* promoter to drive C. albicans *Cas9* (*CaCas9*) expression. We first transformed BsmBI-digested gene-drive plasmids directed against *ADE2* into a wild-type strain and plated cells onto selective medium containing glucose, where the *MAL2* promoter is repressed. Heterozygous integrants (*ade2Δ*::*FRT-MAL2p-CaCas9-FLP-Nat*^*r*^*-sgADE2-FRT*/*ADE2*) were grown in media containing either glucose or maltose and then plated. We found that 30% to 95% of the colonies grown in maltose media displayed an *ade2* phenotype (colony color ranging from light pink to red), whereas less than 1% were red on glucose medium. The rare red colonies on glucose were likely due to leaky expression from the *MAL2* promoter. For both constitutive and inducible Unified Solo vectors, we were able to remove the system by induction of the *FLP* gene, leaving a minimal FRT footprint in the genome and enabling serial mutagenesis.

### CRISPR in C. glabrata, N. castellii, and S. cerevisiae*.*

The design of our *Candida* Solo vector CRISPR system provides advantages for engineering other fungal species, including S. cerevisiae, C. glabrata, and N. castellii. All of these species can maintain episomal plasmids, unlike C. albicans. Most published CRISPR systems for *Saccharomyces* use two distinct plasmids (one to express the sgRNA and the other Cas9) and require selectable auxotrophic markers. The published C. glabrata system also utilizes two plasmids and requires auxotrophic markers (17). These constraints render these systems unusable in prototrophic clinical isolates or industrial strains.

We avoided these constraints by moving our CRISPR Solo insertion into S. cerevisiae vector pRS416, which provides a CEN/ARS element for plasmid maintenance and a *URA3* marker that can be used for counterselection in *ura3* auxotrophs (Fig. 1B and C). We substituted species-appropriate promoter sequences for the sgRNA and CaCas9 to improve their expression. The pRS416 backbone is functional in multiple yeast species, including C. glabrata and N. castellii.

**FIG 4:**
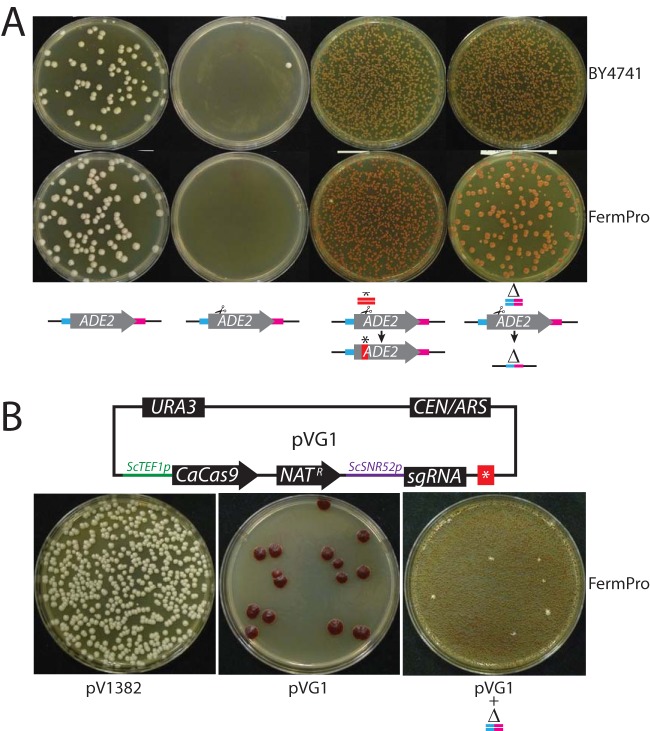
Efficient CRISPR mutagenesis with Solo vectors in S. cerevisiae. (A) Laboratory strain BY4741 and industrial bioethanol strain FermPro were transformed with plasmid pV1382 or plasmid pV1386 with or without the indicated repair templates. (B) FermPro was transformed with Unified Solo vector pVG1 in the presence or absence of a second repair template.

As a proof of principle, we created guides directed against *ADE2* for each of these three species. For S. cerevisiae, we generated *ade2* mutants efficiently only when we included a repair template for creation of either a deletion or a point mutation at *ADE2* (Fig. 4A). No mutants and very few transformants were recovered when the repair template was omitted, presumably because of DSB-induced cell cycle arrest. When a repair template was included, all of the mutants that were recovered contained the sequence encoded by their respective repair templates, indicating that all repair was directed via homology-mediated mechanisms.

To determine if we could consolidate the CRISPR mutagenesis system in *Saccharomyces* as we had for C. albicans (Fig. 4B), we constructed Unified Solo vectors directed against *ScADE2*. Our Unified Solo plasmid efficiently mutagenized *ADE2* without using an integrated gene drive mechanism. These vectors also efficiently edited the genomes of a wide variety of S. cerevisiae strains, including strains used for brewing and industrial fermentations. Addition of an exogenous repair template in the transformation greatly increased the efficiency of mutagenesis, suggesting that repair template is limiting for mutagenesis with Unified Solo vectors (Fig. 4B).

**FIG 5:**
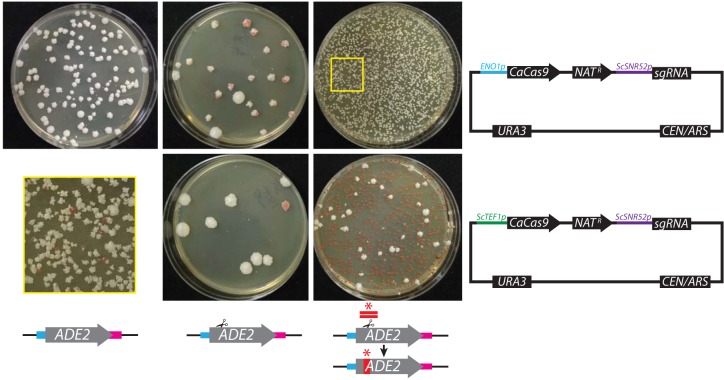
Efficient CRISPR mutagenesis in C. glabrata. Strain BG2 was transformed with pV1326 (top left), pV1329 (top middle), pV1329 with a stop-codon repair template (top right), pV1435 (bottom middle), or pV1435 with a stop-codon repair template (bottom right). The yellow box (bottom left) presents a magnified view of a pV1329 with stop-codon repair transformation plate (top right). Similar results were seen with strain CLIB138. Vectors depict *CaCas9* promoter differences between the two plasmids.

**FIG 6:**
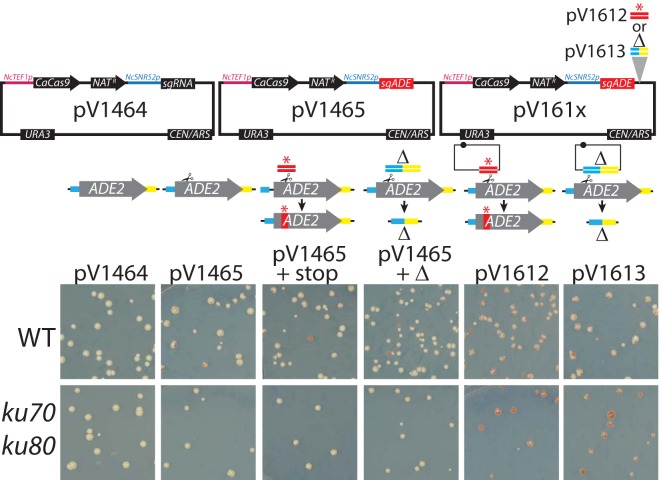
CRISPR mutagenesis in N. castellii. Strain DPB069 (WT) and strain DPB596 (*ku70 ku80*) were transformed with the indicated vectors and repair templates.

Transformation of C. glabrata and N. castellii with a plasmid containing guides for *ADE2* yields *ade2* mutants, both with and without a repair template (Fig. 5 and 6). Moreover, in the absence of a repair template, mutants acquire small deletions or insertions, consistent with repair via an NHEJ pathway. When a repair template was included, 62% and 71% of red *ade2* C. glabrata colonies (Fig. 5, top right and bottom right, respectively) and 7% and 25% of N. castellii colonies (Fig. 6, stop repair versus deletion, respectively) contained the sequence encoded on the repair template, whereas the remaining had indels such as were seen when the repair template was omitted. For C. glabrata, we observed that the choice of *CaCas9* promoter impacted the relative rates of mutagenesis in the presence or absence of a repair template (Fig. 5), similarly to previous observations (17). Mutagenesis with *ENO1p*-expressed *CaCas9* resulted in a higher rate of NHEJ-mediated repair than of templated repair, whereas templated repair predominated for *TEF1p*-expressed *CaCas9*. The rate of mutagenesis seen with a separate repair template was notably lower in N. castellii than in the other species. Consolidation of the repair template onto a Unified Solo vector substantially improved the mutagenesis rate in N. castellii, suggesting that repair template delivery had previously been limiting (56% used a stop repair template, while 69% used a deletion repair template). In N. castellii, we found that simultaneous deletion of the N. castellii orthologues of *KU70* and *KU80*, which are known components of the NHEJ pathway (18, 19), abolished NHEJ repair, as all of the sequenced transformants were shown to have used repair-template-mediated HDR.

In the absence of nourseothricin selection in S. cerevisiae, C. glabrata, and N. castellii, the CRISPR plasmid was lost rapidly (without counterselection), permitting rounds of serial mutagenesis in prototrophic strains. We used this strategy for the serial creation of *met15Δ* mutants and then *leu2* mutants in *C. glabrata* and *ade2* and then *leu2* mutants in S. cerevisiae (data not shown).

### The requirement for a repair template in C. albicans.

C. albicans requires a repair template in addition to Cas9/sgRNA for mutagenesis at a given locus. We hypothesized that this was due to repair of a Cas9 double-stranded break predominantly by the HDR system. To test this directly, we measured *ADE2* mutagenesis in a strain that contained a heterozygous deletion of *ADE2* (*ade2Δ*/*ADE2*). We transformed both wild-type (*ADE2*/*ADE2*) and *ADE2* heterozygotes with an *ADE2*-directed plasmid with and without a repair template containing a base pair substitution that could be distinguished from the deletion. Recovery of *ade2* mutations in the *ADE2*/*ADE*2 wild-type strain required the presence of a repair template, whereas red *ade2*/*ade2* mutants were obtained in the absence of a repair template in the *ade2Δ*/*ADE2* heterozygote (Fig. 2B). Red mutants had only the *ade2Δ*/*ade2Δ* genotype, indicating they either had used the remaining *ade2Δ* allele as a repair template or had become aneuploid for the *ADE2* chromosome or duplicated the *ade2Δ* chromosome through a 2n-1 intermediate. The increased variation in colony size that we observed without the use of an exogenous repair template was similar to results described by Feri and colleagues (20). We were unable to detect any of the indel mutations that are the hallmark of NHEJ-mediated repair. When a repair template was included in the transformation of the *ade2Δ*/*ADE2* heterozygote, approximately 70% of the *ade2* strains used the repair template, whereas the other 30% were genotyped as *ade2Δ*/*ade2Δ*.

### Repair in recombination mutants.

**FIG 7:**
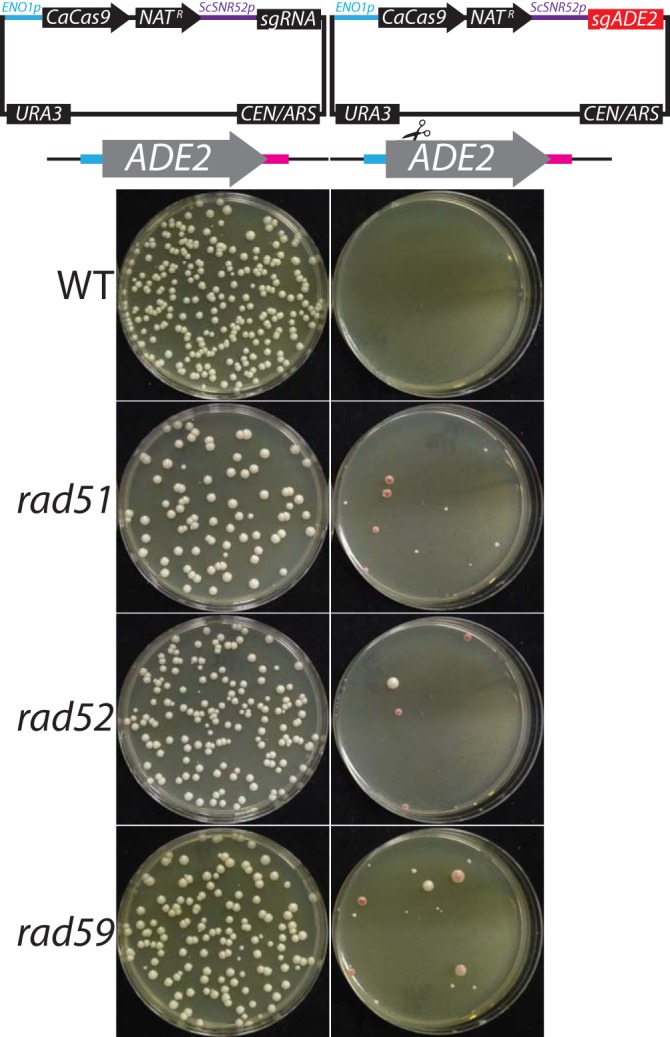
CRISPR mutagenesis in HDR mutants. S. cerevisiae strains of the indicated genotype were transformed with empty vector pV1326 (left) or *ADE2*-targeted vector pV1338 (right) with no repair template.

Our data show that CRISPR mutagenesis requires a repair template in S. cerevisiae and C. albicans, suggesting that HDR is the dominant repair pathway, whereas repair can occur through both the HDR and NHEJ pathways in C. glabrata and N. castellii. We sought to determine whether mutation of the HDR machinery in either S. cerevisiae or C. albicans would increase the frequency of NHEJ-derived mutations when CRISPR targets a locus in the absence of a repair template. To this end, we transformed S. cerevisiae WT (*RAD*), *rad51*, *rad52*, and *rad59* strains with either an untargeted Solo plasmid or an *ADE2*-directed Solo plasmid without a repair template (Fig. 7). As shown previously, we were unable to recover transformants of the WT strain with the *ADE2* guide without the addition of a repair template. However, in strains carrying loss-of-function mutations in *RAD51*, *RAD52*, or *RAD59* genes, we were able to isolate red *ade2* transformants in the absence of a repair template. Sequence analysis revealed that all red *ade2* transformants in the *rad* mutant backgrounds contained indels consistent with NHEJ-mediated repair. The few isolated white colonies contained indels in the *ADE2* sequence that rendered it resistant to further CRISPR cleavage while maintaining *ADE2* prototrophy.

To test if the same strategy would work in C. albicans, we created homozygous mutants in the C. albicans *RAD51*, *RAD52*, *and RAD59* genes (e.g., *rad51*/*rad51*) and tested mutagenesis at *ADE2*. We were unable to generate red *ade2* colonies with our *ADE2*-directed vector in the absence of a repair template in these strains (data not shown).

### Guide selection for C. albicans, C. glabrata, N. castellii, and S. cerevisiae*.*

We designed guide sequences corresponding to each annotated feature in the genomes of C. albicans, C. glabrata, N. castellii, and S. cerevisiae (see the supplemental guide table available at http://osf.io/ARDTX/), using updated guide selection and scoring methods (21), as summarized in Table 1. Most genes are uniquely targetable by multiple guide sequences, and multiple genes can be targeted with single guides (see Text S1 in the supplemental material and the supplemental guide table available at http://osf.io/ARDTX/). For C. albicans, we also designed allele-specific guides using the updated algorithm. Between 77% and 94% of diallelic genes can be uniquely targeted based on these new rules, depending on the off-target algorithm cutoff. In addition, we designed guides and repair templates for use with our (gene-drive-based) Unified Solo vector that link these sequences in *cis* as was done with pV1534. Targeting sequences, chosen from sequences upstream and downstream of each feature, have been screened for the presence of BsmBI sites, since they are required for vector linearization. Guides were also screened to identify those that cut the plasmid backbone. The sequences in these tables simplify cloning design for our Unified Solo vector.

**TABLE 1:** Guide availability in C. albicans, C. glabrata, N. castellii, and S. cerevisiae^a^

Species	No. of unique guides	No. (%) of uniquely targetable genes	Avg no. of unique guides/gene	No. of multigene guides	No. (%) of genes targetable with other genes
C. albicans (old pipeline)	550,045	6,288 of 6,466 (97)	87	105,747	6,023 of 6,466 (93)
C. albicans (new pipeline)	333,464	6,236 of 6,466 (96)	53	12,220	4,647 of 6,466 (72)
C. albicans (allele specific, off-target cutoff of 0.2)	13,286	3,112 of 4,046 (77)	4	NA	NA
C. albicans (allele specific, off-target cutoff of 0.5)	21,765	3,464 of 4,046 (86)	6	NA	NA
C. albicans (allele specific, no off-target cutoff)	39,299	3,805 of 4,046 (94)	10	NA	NA
C. glabrata	197,604	5,285 of 5,597 (94)	38	5,699	2,554 of 5,597 (46)
N. castellii	461,932	5,566 of 5,594 (99)	56	13,208	4,434 of 5,594 (79)
S. cerevisiae	511,439	6,398 of 6,600 (97)	80	14,658	4,522 of 6,600 (69)

a NA, not applicable (allele-specific guides by definition do not target other genes); off-target cutoff score is calculated using software developed by Doench et al. (21). A guide targeting another place in the genome with an off-target cutoff higher than the indicated score is considered unspecific.

## DISCUSSION

Here we designed Solo vectors for CRISPR mutagenesis based on our original system in C. albicans (9), which has also undergone subsequent enhancements and adaptations to other species (10–13, 22). These vectors facilitate a variety of advancements in fungal genome editing. Our refined vectors enable investigators to use an episomal system (C. glabrata, N. castellii, and S. cerevisiae) or to integrate the CRISPR cassette at either a neutral site or the target mutagenesis site (C. albicans); recycle it with a minimal footprint; and/or consolidate the repair template, guide, and Cas9 onto a single molecule. Moreover, these vectors utilize a dominant selectable marker usable in prototrophic clinical isolates and industrial strains.

The redesigned repair templates allow the creation of unmarked kilobase-scale deletions without the use of repeat elements, thus enhancing the efficiency of serial mutagenesis. Though serial mutagenesis has been previously enabled by the use of FLP/FRT-based systems, residual FRT sequences in the genome create opportunities for translocations (23–25), a problem whose solution becomes exponentially more difficult with each subsequent deletion. For our new C. albicans system, we use FRT sequences at only one locus, minimizing the possibility of FLP-mediated translocations. CRISPR-based mutagenesis of members of gene families faces an additional hurdle in that DSB repair of one gene can be templated by other family members. Use of an unmarked deletion repair template addresses both of these issues. Removal of homologous family member sequences with the redesigned repair templates could decrease the likelihood of the occurrence of off-target repairs with each subsequent mutagenesis. Our vectors for C. glabrata, S. cerevisiae, and N. castellii bypass FLP/FRT requirements by virtue of being episomal.

The Solo vectors enabled us to compare results of CRISPR mutagenesis in several different fungal species with a single system based on the same design. CRISPR mutagenesis in C. albicans and S. cerevisiae requires an additional repair template, whereas CRISPR mutagenesis in C. glabrata and N. castellii requires only *Cas9* and guide RNA. These results suggest that the HDR pathway is predominant over the NHEJ pathway in S. cerevisiae and C. albicans and that the NHEJ pathway predominates in N. castellii and C. glabrata. In agreement with this conclusion, mutation of HDR-enabling *RAD* genes bypasses the repair template requirements in S. cerevisiae, leading to repair via NHEJ. Such mutants could potentially simplify and lower the cost of pooled CRISPR screens by eliminating the need to provide a repair template, as well as providing the opportunity to recover a wider range of mutant alleles than can be specified on a repair template. Whereas *RAD* gene mutants bypass the repair template requirements in S. cerevisiae, they do not do so in C. albicans, suggesting that S. cerevisiae and C. albicans have distinct repair preferences.

In C. albicans transformations lacking a repair template, there is almost always a second copy of the gene to provide homology for repair of DSBs. Without a repair template, CRISPR mutagenesis of a strain heterozygous for *ade2Δ*/*ADE2* resulted in homozygosis of the *ade2Δ* allele, either by use of the *ade2Δ* allele as a repair template (gene conversion) or through homozygosis of part or all of the *ade2Δ* chromosome via mitotic recombination or a 2n-1 intermediate. As C. albicans is polymorphic at many loci (designated A and B alleles) (26), this CRISPR mutagenesis would render the strain homozygous for that allele (i.e., A/A or B/B). This feature could be utilized for large-scale LOH studies (to uncover allelic differences) and/or genetic mapping studies, similarly to the concept presented by Sadhu et al. (27). This is particularly useful for C. albicans genetics, as it may circumvent its apparent lack of meiosis that such studies have classically relied upon.

During the preparation of the manuscript, a description of an alternative C. albicans CRISPR gene drive system was published (28). This system was presented as a means to study synthetic genetic interactions, though it could also be used for creating single homozygous knockouts or for creating double homozygous knockouts via mating. In contrast, the architecture of our system allows for multiple gene homozygous knockouts by serial vector recycling and disrupts only the target locus in the genome. Additionally, our inducible vectors represent a novel tool for the creation of conditional mutants.

The Unified Solo vectors provide a straightforward avenue to making deletion libraries in fungal species, especially in those such as C. albicans where manipulation by traditional gene replacement methods has been challenging. Making deletions using the first-generation *Candida* CRISPR vectors required cotransformation of a repair template. Consequently, to make a CRISPR replacement library, repair templates would have to be synthesized for each transformation and cotransformed as a separated arrayed set for any given isolate, as cotransformation of a matched CRISPR/guide module and repair template would be unlikely in a complex pooled library. Our Unified Solo vectors solve this problem by combining the repair template, Cas9, and a guide RNA on a single plasmid that either integrates into the genome at the target mutagenesis site (in the case of C. albicans) or maintains itself as an episome (in the case of S. cerevisiae or N. castellii). Thus, every cell that gets a particular guide RNA also receives its corresponding repair template. Furthermore, libraries of plasmids targeting every locus could be pooled in a single transformation to screen for phenotypes of interest, with guide sequences serving as barcodes to identify a mutant’s abundance (in the case of a pool) or identity (in the case of a clone). The inducible gene drive system permits the creation of on-demand conditional knockouts that can be used for identification of essential genes, pathogenicity factors in hosts, or other developmentally regulated processes. Moreover, each of these systems can recycle the dominant marker, simplifying subsequent mutagenesis. This model of library generation and phenotypic screening has the potential to be broadly applicable.

## MATERIALS AND METHODS

### Transformation.

A hybrid lithium acetate/electroporation protocol was used for C. albicans, S. cerevisiae, and C. glabrata. Briefly, a 5-ml yeast extract-peptone-dextrose (YPD) culture of each strain was grown overnight at 30°C on a roller drum to saturation. Cells were pelleted by centrifugation and resuspended in 2 ml of 100 mM lithium acetate–10 mM Tris (pH 8.0)–1 mM EDTA (pH 8.0) to which 50 µl 1 M dithiothreitol (DTT) was added. These cells were incubated on a 30°C roller drum for 1.5 to 15 h. Next, cells were washed twice in 5 ml of ice-cold water, washed once in 5 ml of ice-cold 1 M sorbitol, and resuspended in 500 µl of ice-cold 1 M sorbitol. Cells were kept on ice while transformation mixes were prepared. Cell suspension (40 µl) was added to aliquoted DNA, mixed by pipetting, placed in chilled Bio-Rad gene pulser cuvettes (Bio-Rad catalog no. 1652086), and electroporated on a Bio-Rad Gene pulser with a capacitance of 25 µF and resistance of 200 Ω at 1.8 kV. YPD medium (100 µl) was immediately added to the cuvette, and the cell mixture is incubated for between 1 and 12 h at room temperature before plating onto appropriate selective media.

N. castellii was transformed using a modified version of the lithium acetate method previously described by Gietz and Schiestl (29). A 5-ml YPD culture of N. castellii was grown overnight at 30°C on a roller drum. This preculture was then used to inoculate 50 ml of YPD medium to reach an optical density at 600 nm (OD_600_) of 0.2. The culture was then grown at 30°C with vigorous shaking in a baffled flask until it reached an OD_600_ of 0.8. Cells were collected by centrifugation, washed once in 25 ml of water, and resuspended in 1 ml of 100 mM LiAc–100 mM Tris-Cl (pH 7.5). This suspension was transferred to a microcentrifuge tube and centrifuged at top speed for 15 s. The pellet was resuspended in 400 µl of 100 mM LiAc–100 mM Tris-Cl (pH 7.5) and incubated at room temperature for 20 min. For each transformation, 50 µl of cell suspension was added to a microcentrifuge tube. To each transformation tube, the following reagents were added in the following order, with vortex mixing performed after each addition: 240 µl 50% polyethylene glycol (PEG) 3350, 36 µl 1 M LiAc, 12.5 µl boiled single-stranded DNA (ssDNA) (10 mg/ml), and 1.5 µg each plasmid DNA and repair template. Transformation mixes were incubated at 23°C for 30 min with rotation and then heat shocked at 37°C for 20 min. Heat-shocked cells were centrifuged at 8,000 rpm for 15 s, and transformation liquid was removed. Cells were washed with 500 µl of water and resuspended in 200 µl of water. At least 25 µl of this suspension was plated onto selective media.

### Strains and growth conditions.

Strains used in this study are listed in Table S2 in the supplemental material. C. albicans strain SC5314, C. glabrata strains BG2 and CLIB138, S. cerevisiae strains BY4741 and FermPro, and N. castellii strains DBP069 and DBP596 were used for experiments. Yeast cells were grown in YPD medium (1% Difco Bacto yeast extract, 2% Difco Bacto peptone, 2% glucose) and selected with nourseothricin (Nat) at between 100 and 200 µg/ml. Flipout of the Nat^r^ gene from C. albicans vectors was induced by overnight growth in Difco yeast carbon base supplemented with bovine serum albumin (4%) overnight or by overnight growth in YP maltose medium (1% Difco Bacto yeast extract, 2% Difco Bacto peptone, 2% maltose). Episomal plasmid loss experiments were performed by overnight growth in nonselective liquid YPD medium. Drug-sensitive isolates, which had either flipped out the cassette or lost the plasmid, were identified by plating for single colonies on nonselective media and subsequent identification by replica plating to selective media.

Filamentation experiments were performed with yeast grown overnight in liquid YPD medium, washed twice with RPMI 1640 medium (Thermo Fisher catalog no. 22400-105) supplemented with 10% fetal bovine serum (FBS), and incubated in RPMI medium–10% FBS for the indicated time starting at an optical density of 0.1. CRISPR-mutagenized loci were verified by agarose gel electrophoresis and sequencing of PCR products amplified from target loci.

### Plasmids/DNA.

Plasmids for *CaCas9* system vectors are listed in Table S1. All key components were verified by sequencing and restriction analysis. Vectors and sequences will be available on Addgene. Transformations were performed with between 0.3 and 5 µg of plasmid DNA (smaller amounts were used for episomal plasmids and larger amounts for linear vectors for integration) and with between 1 and 5 µg of a repair template (where applicable). Repair templates were generated with 60-bp oligonucleotide primers containing 20-bp overlaps at their 3′ ends centered at the mutation point, which consisted of either stop codons or an open reading frame (ORF) deletion. N. castellii repair templates were 500 bp in length. Oligonucleotide sequences are listed in Table S3. Primers were extended by thermocycling performed with *Ex Taq* (TaKaRa catalog no. RR001A), and double-stranded products were purified directly from the PCR using a QIAquick gel extraction kit with isopropanol. Phosphorylated and annealed guide sequence-containing primers were ligated into calf intestinal phosphatase (CIP)-treated, BsmBI-digested parent vectors as described previously (9). The correct clones were identified by sequencing.

10.1128/mSphere.00154-18.3 TABLE S1  Plasmids used in this study. Download TABLE S1, PDF file, 0.1 MB. Copyright © 2018 Vyas et al. 2018 Vyas et al. This content is distributed under the terms of the Creative Commons Attribution 4.0 International license.

10.1128/mSphere.00154-18.4 TABLE S2  Strains used in this study. Download TABLE S2, PDF file, 0.1 MB. Copyright © 2018 Vyas et al. 2018 Vyas et al. This content is distributed under the terms of the Creative Commons Attribution 4.0 International license.

10.1128/mSphere.00154-18.5 TABLE S3  Oligonucleotides used for repair template generation. Download TABLE S3, PDF file, 0.05 MB. Copyright © 2018 Vyas et al. 2018 Vyas et al. This content is distributed under the terms of the Creative Commons Attribution 4.0 International license.

### Computational analysis for guide design.

We designed guide sequences corresponding to each annotated gene in the genomes of C. albicans, C. glabrata, S. cerevisiae, and N. castellii (see the supplemental guide tables [DOI https://doi.org/10.17605/OSF.IO/ARDTX] available at http://osf.io/ARDTX/), using updated guide selection and scoring methods (21) (summarized in Table 1).

For C. glabrata, S. cerevisiae, and N. castellii, we listed all 20 nucleotides (nt) followed by NGG (or CCN plus 20 nt) in the genome. We removed any targets that had 6 instances of "T" in the 20 nt before the NGG because their presence would result in premature termination from polymerase (Pol) III promoters. We kept only the guides that were hitting the coding regions of genes. To find putative off-targets, we took the 14 nt closest to the protospacer-adjacent-motif (PAM) sequence, constructed the four possible sequences of the PAM, and mapped these with Bowtie, allowing up to 3 mismatches. For each original guide, we calculated the on-target score using rule set 2 for sgRNA on-target activity script (described in reference 21) as well as all the possible off-target scores for all the places in the genome that the 14-nt NGG hit with ≤3 mismatches (cutting frequency determination [CFD] scoring) (21). We defined guides as having no off-targets if the off-target scores at other locations were lower than 0.2. To find guides that targeted more than one gene, we followed the requirements that the off-target score for the other genes hit was 0.5 or higher and that there were no other hits with off-target scores higher than 0.2 and lower than 0.5. We also removed guides with off-targets in intergenic regions.

For C. albicans, using rule set 2 for the sgRNA on-target activity script (21), we rescored the guides uniquely targeting the two alleles that we had previously designed (9). Additionally, to assess whether those guides could be used on the Unified Solo vector, we checked that the guide was not targeting the vector and the absence of BsmBI sites in the guide.

Sequences (FASTA files) and annotations (GFF files) for C. albicans and C. glabrata were downloaded from the Candida Genome Database (30). C. albicans sequences from strain SC5314 (assembly 22, version A22-s05-m01-r03) and C. glabrata sequences from strain CBS138 (version s02-m07-r06) were used for guide selection. N. castellii guide design was based on sequences (FASTA files) and annotations (GFF files) for strain CBS4309 (assembly ASM23724v1.31) obtained from Ensembl (31). S. cerevisiae guide design was based on sequences (FASTA files) and annotations (GFF files) downloaded from the Saccharomyces Genome Database (32). The sequences were from strain S288C (version sacCer3/SGD_2010).

### Data availability.

Species-specific guide sequence tables (for single-gene, multigene, and/or allele-specific guides) are available at the Open Science Frameworks repository (DOI https://doi.org/10.17605/OSF.IO/ARDTX; https://OSF.IO/ARDTX).
