# Supplementary figures and images for: New CRISPR Mutagenesis Strategies Reveal Variation in Repair Mechanisms among Fungi
Source: mSphere. 2018 Apr 25;3(2):e00154-18. doi: 10.1128/mSphere.00154-18 (PMC5917429; doi:10.1128/mSphere.00154-18)

A

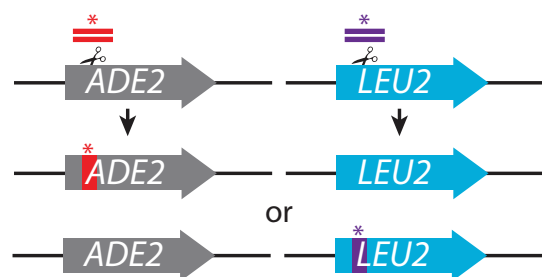

YPD → SC -Leu replica

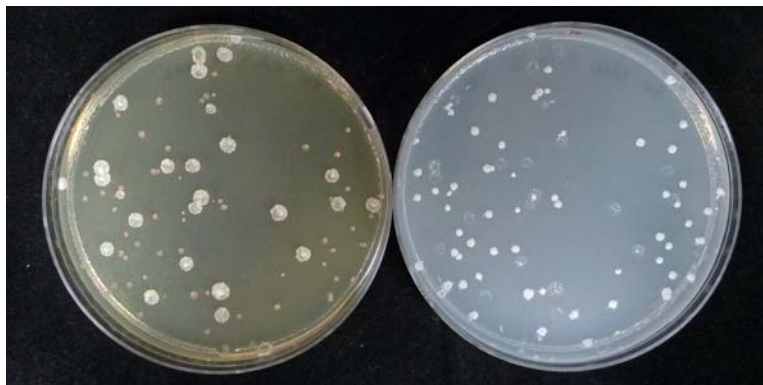

B

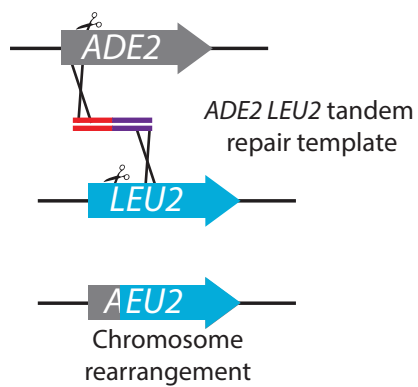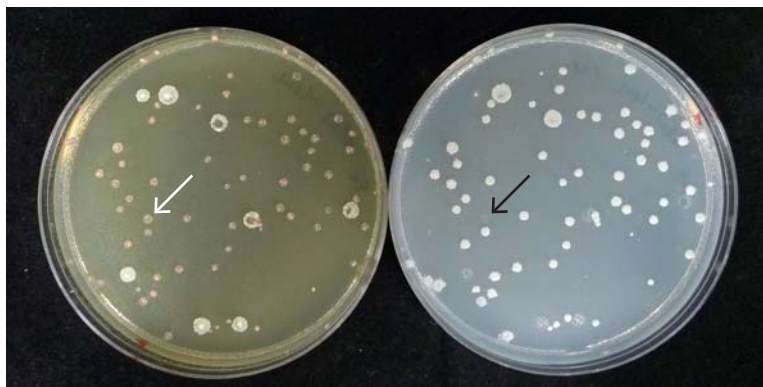

C

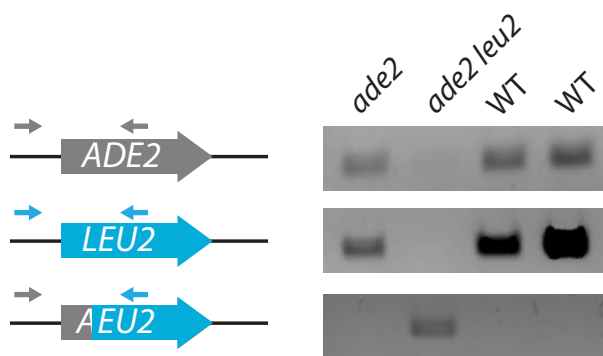

Supplement: FIG S1 [file sph002182526sf1.pdf]
