# Supplementary material for: New CRISPR Mutagenesis Strategies Reveal Variation in Repair Mechanisms among Fungi
Source: mSphere. 2018 Apr 25;3(2):e00154-18. doi: 10.1128/mSphere.00154-18 (PMC5917429; doi:10.1128/mSphere.00154-18)
Supplement: TEXT S1 [file sph002182526s1.pdf]

Targeting multiple genes in one transformation using CRISPR

We tested if we could use our CRISPR system to target multiple genes in a single transformation. Two approaches were compared: transformation of two plasmids targeting two different loci along with two separate repair templates, or transformation of two plasmids targeting two different loci but with a single "fused" repair template that contains homology to both loci in tandem (Figure S1). We chose *ADE2* and *LEU2* as our targets. Transformation with plasmids and repair templates that each target *ADE2* and *LEU2* individually results in mutants that are either adenine auxotrophs or leucine auxotrophs, but not both (Figure S1A). Transformation with plasmids either against *ADE2* and *LEU2* along with a tandem *ADE2-LEU2* repair template resulted mostly in mutants that are either adenine auxotrophs or leucine auxotrophs, but with some colonies of transformants with both double adenine and leucine auxotrophies (Figure S1B).

We hypothesized that the use tandem repair templates might result in chromosomal rearrangements if the template is used to bridge break sites on two different chromosomes (Figure S1B). To test this idea, we performed a PCR on *ade2* and *leu2* mutants from a transformation with plasmids carrying guides against *ade2* and *leu2*, and a tandem repair template that inserts premature stops in *ADE2* and *LEU2*. Three primer pairs were used to amplify either *ADE2*, *LEU2*, or a potential *ADE2-LEU2* fusion, if present. *ade2* mutants and *leu2* mutants contained the ORF, as expected, since the repair template causes encodes insertion of premature stop codons. However, amplification of *ADE2* or *LEU2* failed in *ade2 leu2* double mutants, while a product corresponding to an *ADE2-LEU2* fusion was detected (Figure S1C), suggesting that our fusion repair template was leading to genomic rearrangement linking these ORFs during the repair process.
