# Supplementary material for: New CRISPR Mutagenesis Strategies Reveal Variation in Repair Mechanisms among Fungi
Source: mSphere. 2018 Apr 25;3(2):e00154-18. doi: 10.1128/mSphere.00154-18 (PMC5917429; doi:10.1128/mSphere.00154-18)
Supplement: TABLE S1 [file sph002182526st1.pdf]

Plasmid	Description
pV1393	*C. albicans* Solo CRISPR vector flanked by Neut5+FRT targeting regions; FLP expression driven by *SAP2* promoter
pV1524	*C. albicans* Solo CRISPR vector flanked by Neut5+FRT targeting regions; FLP expression driven by *MAL2* promoter
pV1539	*C. albicans* Gene Drive vector - derived from pV1524 Kpn1/Xma1 backbone replaced with Kpn1/Xma1 fragment from pV1093
pV1543	*C. albicans* Gene Drive vector - derived from pV1539 with second Sac2 site placed between BsmBI sites
pV1326	*S. cerevisiae* and *C. glabrata* Solo CRISPR vector, marked with *URA3* and Nat^R
pV1338	pV1326 + sgScADE2
pV1382	*S. cerevisiae* and *C. glabrata* Solo CRISPR vector, marked with *URA3* and Nat^R
pV1386	pV1382 + sgScADE2
pV1464	*N. castellii* Solo CRISPR vector, marked with *URA3* and Nat^R
pVG1	pV1382 + sgScADE2 + ScADE2 stop codon repair tempate
pV1534	*C. albicans* Gene drive vector for *ADE2,* with BsmBI *in vitro* linearization sites
pV1535	*C. albicans* Gene drive vector for *ADE2* with sgADE2 *in vivo* linearization site
pV1418	pV1393 + sgCPH1
pV1420	pV1393 + sgEFG1
pV1528	pV1524 + sgCaLEU2
pV1531	pV1524 + sgCaMET15
pV1487	pV1382 + sgCgMet15
pV1518	pV1382 + sgCgLeu2
pV1435	pV1382 + sgCgADE2
pV1329	pV1326 +sgCgADE2
pV1465	pV1464 + sgNcADE2
pGB9	pV1382 + sgScLEU2
pV1563	*C. albicans* Inducible Gene Drive vector - *MAL2* driven *CaCas9, SAP2-FLP* (with intron) - second Sac2 site for cloning
pV1571	*C. albicans* maltose-inducible Gene Drive vector for *ADE2* , pV1563 + guide/targeting/linearization regions as in pV1534
pV1465	pV1464 with sgNcADE2
pV1612	pV1465 with stop repair template
pV1613	pV1465 with delta repair template
