# Supplementary material for: New CRISPR Mutagenesis Strategies Reveal Variation in Repair Mechanisms among Fungi
Source: mSphere. 2018 Apr 25;3(2):e00154-18. doi: 10.1128/mSphere.00154-18 (PMC5917429; doi:10.1128/mSphere.00154-18)
Supplement: TABLE S2 [file sph002182526st2.pdf]

Strain	Genotype	Background
Candida albicans		
SC5314		
VY987	*efg1Δ/efg1Δ Neut5L::FRT/Neut5L*	SC5314
VY989	*cph1Δ/cph1Δ efg1Δ/efg1Δ Neut5l::FRT/Neut5L*	SC5314
VY1024	*leu2Δ/leu2Δ met15Δ/met15Δ Neut5L::FRT/Neut5L*	SC5314
VY1036	*ade2Δ::FRT-CaCas9-FLP-Nat*^{R}*-sgADE2-FRT (from pV1535)/ade2Δ::FRT-CaCas9-FLP-NatR-sgADE2-FRT (from pV1535)*	SC5314
VY1037	*ade2Δ::FRT-CaCas9-FLP-Nat*^{R}*-sgADE2-FRT (from pV1532)/ade2Δ::FRT-CaCas9-FLP-NatR-sgADE2-FRT (from pV1532)*	SC5314
VY1043	*ade2Δ::FRT-MAL2p-CaCas9-FLP-Nat*^{R}*-sgADE2-FRT (from pV1571)/ADE2*	SC5314
Saccharomyces cerevisiae		
BY4741	*MatA his3Δ1 leu2Δ0 met15Δ0 ura3Δ0*	S288C
L6437	*MatA his3Δ::hisG leu2Δ::hisG ura3-52*	Σ1278b
FermPro	Industrial bioethanol strain	Ferm Solutions
Candida glabrata		
BG2		
VY1021	*met15Δ/met15Δ*	BG2
VY1028	*met15Δ/met15Δ leu2/leu2*	VY1028
CLIB138		
Naumovozyma castellii		
DPB069	*MatA ho leu2 ura3-1 his3 lys2*	
DPB596	*MatA ho leu2 ura3-1 his3 lys2 ku70 ku80*	DPB069
