# Supplementary material for: New CRISPR Mutagenesis Strategies Reveal Variation in Repair Mechanisms among Fungi
Source: mSphere. 2018 Apr 25;3(2):e00154-18. doi: 10.1128/mSphere.00154-18 (PMC5917429; doi:10.1128/mSphere.00154-18)
Supplement: TABLE S3 [file sph002182526st3.pdf]

Primer name	Sequence
CaADE2-delta-fwd	caagcactacacataatggatagcaaaactgttggtattttaggaggtggctgataaatt
CaADE2-delta-rev	ttaatatgctattgatatctatatttttttctatttttcaatttatcagccacctccta
CaMet15-delta-fwd	TTTTGTTTTAATTCTTCCTTTTTTGTTTTCCCCATTACTAAACTACAATAGATTGGT
CaMet15-delta-rev	TTAATGTGTCTAATTATTGTTGTGAAACGTTATCTTATATACCAATCTATTGTAGTT
CaLeu2-delta-fwd	TCTTGAAAGCAATTGAAGCTGCCACTCCCTACCAAAAAATCCAATTTGATACAGTATATA
CaLeu2-delta-rev	GTAGACTTTGGAGTATTTGAAATTTAAATGCTAACTACTGTATATACTGTATCAAATTGG
CaCph1-delta-fwd	GTAGACTTTGGAGTATTTGAAATTTAAATGCTAACTACTGTATATACTGTATCAAATTGG
CaCph1-delta-rev	CATTTACTTAGTTTTTTTCTTTCTTTCTCTTTCTCTCTGTATCTATTCATGGCGAAAGAG
CaEfg1-delta-fwd	AACGAATTAAGATTTGTTCTATTTGACTACCAAGAATATAACCCATATTATAAATATCAT
CaEfg1-delta-rev	TTTGGAATTTATGGCAGAAAGCAGAAGGTGATGTACACAAATGATATTTATAATATGGGT
ScADE2-stop-fwd	ATGGATTCTAGAACAGTTGGTATATTAGGAGGGGGACAATTGGGACGTATGATTtaatga
ScAde2-stop-rev	GTATTACCGTCTTAATGTTGAGCCTGTTTGCTGCgaattctcattaAATCATACGTCCCA
ScAde2-delta-fwd	AAACAATCAAGTATGGATTCTAGAACAGTTGGTATATTAGGAGGGGGACATATATAAGTT
ScAde2-delta-rev	ATATCATTTTATAATTATTTGCTGTACAAGTATATCAATAAACTTATATATGTCCCCCTC
ScLeu2-stop-fwd	AGAAGATCGTCGTTTTGCCAGGTGACCACGTTGGTCAAGAAtaatagGaattcGCCATTA
ScLeu2-stop-rev	TTGACATTGGAACGAACATCAGAAATAGCTTTAAGAACCTTAATGGCgaattCctattaT
Ade-leu-tandem-fwd	ATGGATTCTAGAACAGTTGGTATATTAGGAGGGGGACAATTGGGACGTATGATTtaatgagaattcGCAGCAAACAGGCTCAACATTAAGACGGTAATACAGAAGATCGT
ade-leu-tandem-rev	TTGACATTGGAACGAACATCAGAAATAGCTTTAAGAACCTTAATGGCgaattCctattaTTCTTGACCAACGTGGTCACCTGGCAAAACGACGATCTTCTGTATTACCGT
CgAde-stop-fwd	ATGGACTCTAGAACTGTCGGTATTTTGGGTGGAGGCCAGTTGGGCCGTATGATTGTCTAA
CgAde-stop-rev	CAAGTATCAAAGTCTTGATATTCAAGCGGTTTGCAGAAGCTTAGACAATCATACGGCCCA
CgLeu-delta-fwd	AAGTTTTAAATGCTATTCAGGAATGTCGTCCAGACAAGGTCAATTTCAAGACTTAGTAGA
CgLeu-delta-rev	TTTACTTCTTTCTGTTCAGACTTATGTATGCAACTGTACATCTACTAAGTCTTGAAATTG
CgMet-delta-fwd	GGAGAACCCTGACGACAACAACCACAGACCTAGAGCTGTGCCAATCTACTGTATAGTAAA

CgMet-delta-rev	GTTTACATTGGCATACATATTTGCATGCTTGGTATGTGCTTTTACTATACAGTAGATTGG
NcAde-stop-fwd	GACGTCCGACTTTTCTGACTCTTTTATTTCGCTTATCTTGACTTCTCTGAAAAATAATGGATATTTTTAAGAACATTTCAAGAACATACCTCTCTATATAATGTCAAAACTTTTTGACTTCTTTCATCGCTATTCTCACAATTGTAGGTCATATATAAGAAGCTCATCTGCATCTTAGATACAAACCTAACGATAAAAGCAATAATAGCAGCAAAGCAACAATAATGGATCAAAGAATAGTCGGTATATTAGGGGGTGGACAACTAGGCAGAtaatgagaattcGAA
NcAde-stop-rev	ACTGAATTGTCTCCGGAGTTGGATATATTTTCAAATTTGGATAAGTTAATTGCACACTTTGCAACGTCAACACATCCACATGTTCAATCTCGATAGTCAAAACATCACACTTAGCAGCTAACTTTTCAATATCTTGTGGGTTAGTGTACGATCCATCGACATGTTCAGAGCTGTTACTAATTTGCTTCGCTGGAGAGTTTGGAGCATCAAGTACGATAGTACGTATATTCAAACGATTGGCCGCTTCgaattctcattaTCTGC
NcAde-delta-fwd	GACGTCCGACTTTTCTGACTCTTTTATTTCGCTTATCTTGACTTCTCTGAAAAATAATGGATATTTTTAAGAACATTTCAAGAACATACCTCTCTATATAATGTCAAAACTTTTTGACTTCTTTCATCGCTATTCTCACAATTGTAGGTCATATATAAGAAGCTCATCTGCATCTTAGATACAAACCTAACGATAAAAGCAATAATAGCAGCAAAGCAACAATAATTGCCTTAT
NcAde-delta-rev	GGGATATTATCGATATGAAAGAAAGAAATTATATAGTGGGATTCATTTTTGCAAACTATAAACGTCTCATATAGACAGGAAACGATACAACTAATGAAACAACCACTTTACCCAGGATACTAAATATAACATGACTGGGGAAGTTGTATAAATACAATATATTACTATTTACGAACTCTATAAAATTCTATTAATTCTTTGTGTTTATAGAGAAGGCATACATATCAGATGCTTACTTTCATAAGGCAATTATTGTTGCT
